# High-Performance Methylsilsesquioxane Aerogels: Hydrolysis Mechanisms and Maximizing Compression Properties

**DOI:** 10.3390/gels9090720

**Published:** 2023-09-05

**Authors:** Guihua Zhang, Chengdong Li, Yuxiang Wang, Liangliang Lin, Kostya (Ken) Ostrikov

**Affiliations:** 1 Key Laboratory of Synthetic and Biological Colloids, Ministry of Education, School of Chemical and Material Engineering, Jiangnan University, Wuxi 214122, China; 2 International Joint Research Center for Photoresponsive Molecules and Materials, School of Chemical and Material Engineering, Jiangnan University, Wuxi 214122, China; 3 School of Chemistry and Physics and QUT Centre for Materials Science, Queensland University of Technology (QUT), Brisbane, QLD 4000, Australia

**Keywords:** methylsilsesquioxane aerogels, hydrolysis, formation mechanisms, microstructure, compression strength

## Abstract

Synthesis of methylsilsesquioxane aerogels by ambient pressure drying instead of supercritical drying has recently emerged as a major trend, but the issues of low mechanical strength and unstable performance still need to be resolved. This work reveals the microscopic formation mechanisms of gel skeleton based on the kinetic characteristics of methyltrimethoxysilane (MTMS) precursor hydrolysis and the associated sol-gel reactions. The effects of oxalic acid concentration (*c*_OA_) and hydrolysis time of MTMS solution (*t*_h_) on the gelation time, morphologies, microstructures, chemical structure, and compression properties of the as-synthesized methylsilsesquioxane aerogels are investigated. The optimal *c*_OA_ and *t*_h_ are 38.4 mmol/L and 120 min, respectively, endowing the methylsilsesquioxane aerogels with a compression strength of 0.170 MPa and a maximum compression strain of 61.2%. Precise control of the hydrolysis conditions ensures the formation of branched particle-to-particle networks, which is crucial for maximizing the compression properties of methylsilsesquioxane aerogels synthesized under industry-relevant conditions.

## 1. Introduction

Silica aerogels are nanoporous silica materials synthesized by a silica precursor’s sol-gel and unique drying process so that the gas replaces the liquids within the gel pores while the gel skeleton remains intact [1,2]. Their remarkable properties [3,4,5], such as low density (typically 30–180 mg/cm^3^), high porosity (80–99.9%), high surface area (500–1200 m^2^/g), ultra-low thermal conductivity (~15 mW/(m·K)), and high mesopore volume (1–5 cm^3^/g), make silica aerogels attractive for versatile applications, including thermal insulation [6], fire protection [7], space exploration [8], CO_2_ capture [9], oil-water separation [10], and others. In particular, silica aerogels can improve the high-temperature tolerance and low-temperature performance of electrochemical batteries while preventing unwanted thermal runaway effects due to their superinsulation property and high thermal stability (>400 °C) [11,12,13]. In recent years, the emerging markets in the thermal management of electric vehicles have driven the large-scale applications of silica aerogel products [14,15]. The global aerogel market was valued at USD512.9 million in 2016 and is estimated to reach USD8083.7 million in 2026, with a compound annual growth rate of 31.8% [16]. To satisfy the application requirements, improving the overall performance and shortening the preparation time of the silica aerogels are thus necessary [17].

High and stable mechanical properties are the preconditions for further expansion of aerogel applications. One approach for solving the silica aerogels’ inherent brittleness and poor mechanical properties is to employ trialkoxysilanes, such as methyltrimethoxysilane (MTMS), as precursors to synthesize methylsilsesquioxane (MSQ, CH_3_SiO_1.5_) aerogels with nanoporous networks [18,19,20]. The underlying reason for this is the “spring-back” effect from the organic-inorganic hybrid network consisting of numerous methyl (-CH_3_) groups and the lower cross-linking density of Si-O-Si bonds. This unique network endows the MSQ aerogels with higher mechanical strength, flexibility, and resilience [21]. Lei et al. [22] found that the supercritical-dried MSQ aerogel possessed a robust texture that could withstand high compression stress of ~0.35 MPa. In contrast, the ambient-dried MSQ aerogels possess a much lower compression strength, i.e., ~0.06 MPa, as reported in Ref. [20], which is needed more for practical applications. Compared with currently dominant supercritical drying (SCD), ambient pressure drying (APD) presents several advantages, such as extra safety (no need for high-pressure equipment) [23,24] and energy-efficient features [25], which is a promising alternative for mass production of silica aerogels. However, only a few pieces of literature focused on the strategies for improving the mechanical properties of ambient-dried MTMS-derived MSQ aerogels.

**Scheme 1:**
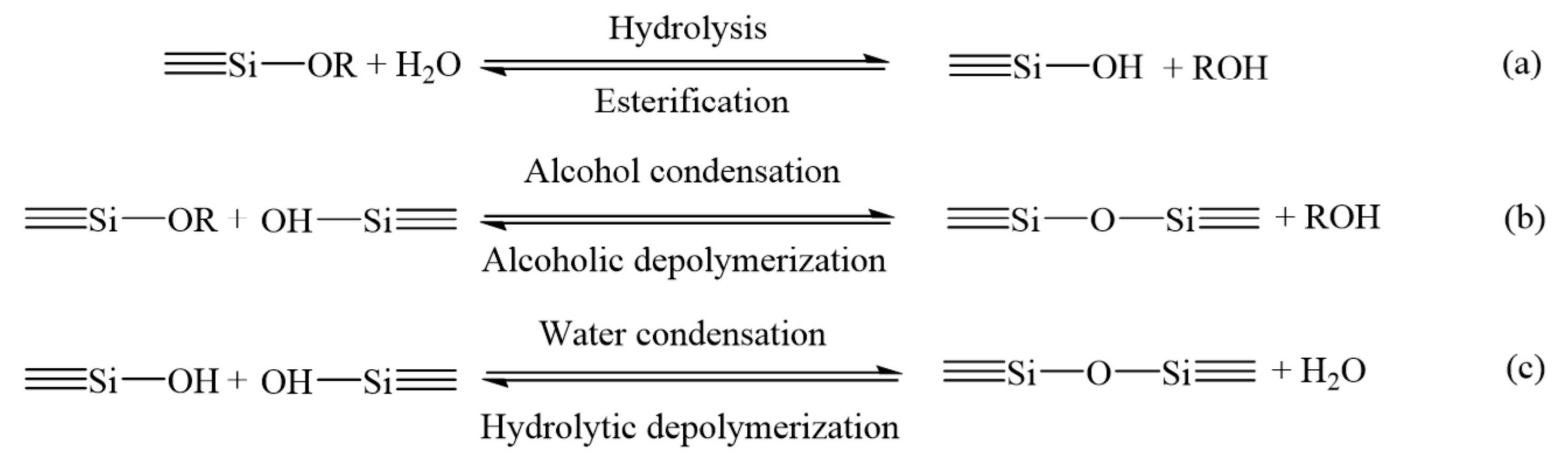
Hydrolysis and condensation reactions of MTMS in the sol-gel process.

The preparation of silica aerogels is divided into two steps [26,27]: (a) the sol-gel process that converts precursor solution into sol and wet gel through hydrolysis and condensation reactions (see Scheme 1), and (b) the drying of wet gel. The basic chemical principle behind sol–gel processing of the MTMS precursor is the transformation of silanol (Si-OH) and methoxy (Si-OCH_3_) containing species to siloxane (Si-O-Si) compounds by condensation reactions. From a structural point of view, this corresponds to connecting CH_3_SiO_3_ tetrahedra in the gel materials by corner sharing. A sufficient number of Si-O-Si bonds and minimized number of Si-OH and Si-OCH_3_ groups has to be obtained in order to produce a stable and robust aerogel. Although the hydrolysis and condensation reactions of MTMS occurred concurrently, the hydrolysis rate was greater than the condensation rate of the MTMS solution under the acidic conditions, while vice versa under the alkaline conditions [28,29]. Therefore, the acid/base two-step catalyzed sol-gel process is generally adopted for the synthesis of MSQ aerogels.

**Figure 1:**
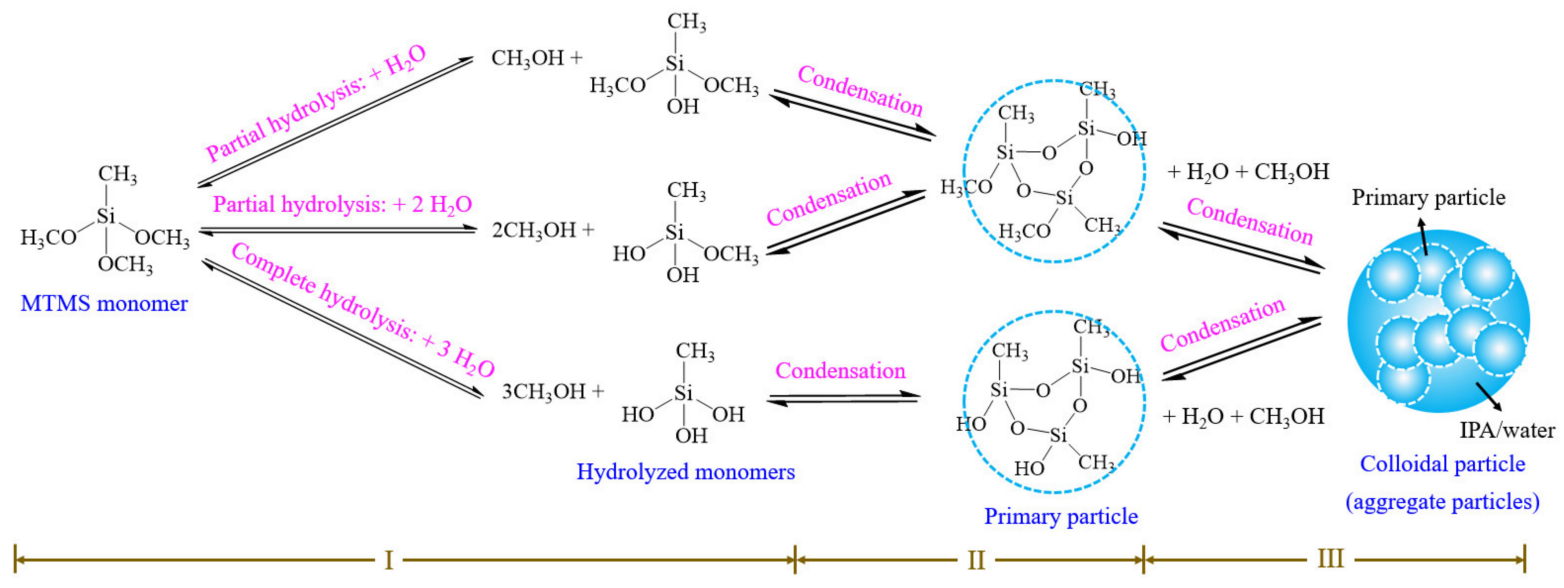
Chemical reactions occurring in the hydrolysis of MTMS.

In the first step of the acid/base two-step catalyzed sol-gel process, the hydrolysis reactions of Si-OCH_3_ groups must precede condensation (see Scheme 1a) in order to generate the Si-OH groups that are necessary for condensation. In the second step, the condensation (i.e., formation of Si-O-Si units) takes place by either alcohol and water elimination (see Scheme 1b,c). The hydrolysis of silica precursor solution, which involves ionic reactions for polymerization, is one of the decisive stages determining the final product properties [30]. For instance, each MTMS monomer (see Figure 1) had one non-hydrolyzable -CH_3_ group and three hydrolyzable methoxy groups (-OCH_3_). As reported in Refs. [31,32], the hydrolysis of MTMS precursor solutions proceeds in a stepwise manner. The process steps involved are: (I) the conversion of MTMS to silanols (Si-OH), (II) the condensation of silanol (Si-OH) to siloxane (-Si-O-Si-) bonds linked primary particles, and (III) the coalescence (i.e., growth and branching) of primary particles into MTMS colloidal particles [26,33]. Completely hydrolyzed monomers (i.e., CH_3_Si(OH)_3_) and partially hydrolyzed monomers (i.e., CH_3_Si(OCH_3_)(OH)_2_ and CH_3_Si(OCH_3_)_2_(OH)) were formed under various hydrolysis conditions [20]. As the condensation progresses, arising from the phase separation of MSQ and water, colloidal particles with sea-island structures are formed.

Previous reports have demonstrated that the hydrolysis parameters, such as the H_2_O/Si molar ratio [34], pH [35,36], and nature and concentration of catalyst [37,38], profoundly affect the structures and properties of as-synthesized sol-gel silicates. Apart from optimizing the starting composition of silica precursors, the hydrolysis time (*t*_h_) of the silica precursor and concentration of acid catalyst is expected to affect the hydrolysis degree of the silica precursor and, thereby, the morphology and properties of as-synthesized MSQ aerogels. However, even for the same silica precursor, such as MTMS, the reported *t*_h_ values vary greatly, ranging from 0.5 h [39] to one day [40]. A low concentration of acid catalyst in the sol or insufficient *t*_h_ could cause incomplete hydrolysis of MTMS. Therefore, optimizing the hydrolysis conditions for silica aerogels is thus critical to ensure their high performance and further reduce their synthesis time and energy cost. There is a clear knowledge gap in the correlation between the dominant hydrolysis kinetics of silica precursors with the properties and microstructure of the synthesized silica aerogels.

Herein, we report a facile acid/base two-step catalytic sol-gel and APD process to prepare highly robust MSQ aerogels from MTMS precursor. Instead of the most common methanol (with a surface tension of 24.0 mN/m at 20 °C [41]), isopropyl alcohol (IPA) was used as the solvent for homogenizing MTMS due to its lower surface tension (22.8 mN/m at 20 °C [42]). Besides, since IPA does not engage in the hydrolysis and condensation reactions of MTMS, it will not inhibit the hydrolysis reactions and alcohol condensation reactions of siloxanes (Si-OR). Robust MSQ aerogels with a more compact Si-O-Si network skeleton can thus be synthesized through the sufficient hydrolysis and condensation reactions of MTMS. In this study, the influence of oxalic acid concentration in the sol (*c*_OA_) and *t*_h_ on the gelation time (GT), macroscopic morphologies, bulk densities (*ρ*), linear shrinkage ratios (*χ*), particle sizes (*d*), microstructures, and compression properties of the obtained MSQ aerogels are investigated. The overall properties of as-synthesized MSQ aerogels are optimized based on the hydrolysis kinetics. The aims of this paper are to provide new perspectives into the hydrolysis mechanisms of MTMS precursors and their impact on the properties of the associated wet gels and aerogels in order to enhance the rapid preparation of silica aerogels featuring high mechanical strength and stable properties under industry-relevant conditions.

## 2. Results and Discussion

### 2.1. Hydrolysis Kinetics of MTMS Solutions

**Figure 2:**
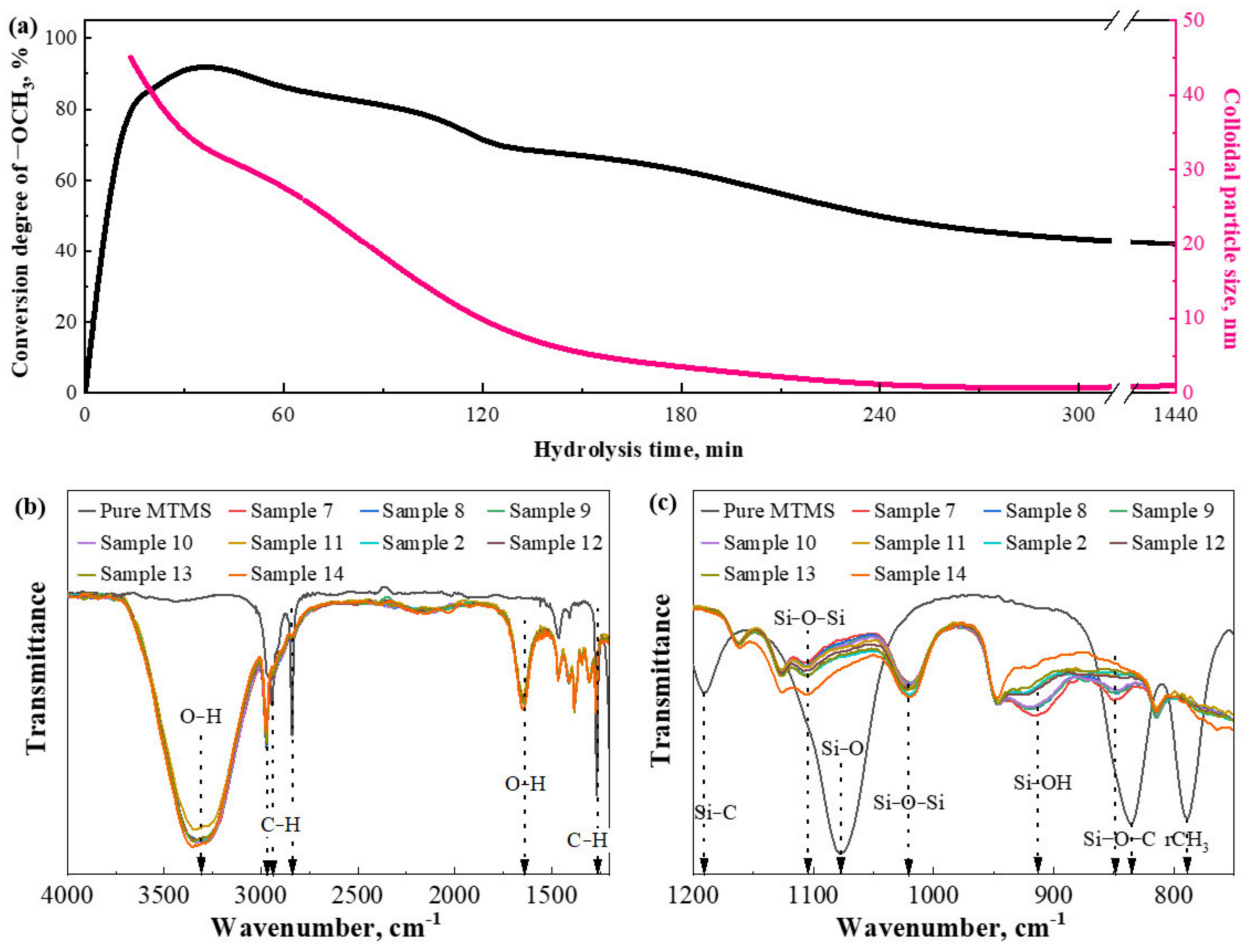
(**a**) Conversion rate of -OCH_3_ of the MTMS solution catalyzed by the OA aqueous solution (*c*_OA_ = 38.4 mmol/L) and colloidal particle size within the MTMS sol as a function of *t*_h_. Notes: the conversion degree of −OCH_3_ (*η*) quantifies the hydrolysis degree of MTMS solutions, which was calculated by Equation (1). The colloidal particle sizes within the MTMS solution at various *t*_h_ were determined by the static and dynamic light scattering instrument (ALV/CGS-3, ALV-Laser Vertriebsgesellschaft m-b.H., Langen, Germany) with a fixed scattering angle of 90°. FTIR spectra of as-synthesized sol samples with various *t*_h_: (**b**) wavenumber of 4000–1200 cm^−1^, and (**c**) 1200–750 cm^−1^. Notes: The *t*_h_ for sol Samples 7–11, 2, and 12–14 was 10 min, 30 min, 45 min, 60 min, 105 min, 120 min, 135 min, 180 min, and 1440 min, respectively.

The hydrolysis rate of MTMS precursor is much higher than the polycondensation rate in the acidic medium. In this work, the kinetics and degree of hydrolysis reactions were tailored by the additional amount of OA aqueous solution and *t*_h_. From Scheme 1a, each hydrolysis reaction of Si-OCH_3_ produced one Si-OH moiety and one methanol (CH_3_OH) molecule. Hence, the GC technique was used in this paper to monitor the methanol content produced by the hydrolysis of MTMS monomer to obtain the conversion rate of -OCH_3_. The hydrolysis time is the time of magnetic stirring MTMS solution at 400 rpm after the addition of deionized water and OA aqueous solution into the MTMS solution. As shown in Figure 2a, the conversion rate of -OCH_3_ increased remarkably immediately after the addition of OA aqueous solution and reached the maximum value of 92.6% after hydrolysis for 30 min. The generated Si-OH moieties would prefer further crosslinking (see Scheme 1b,c) in the subsequent condensation reactions [32]. The reasons for the decrease in the conversion rate of -OCH_3_ after *t*_h_ exceeded 30 min can be attributed to two factors. On one hand, no more methanol was produced as the hydrolysis reactions (see Scheme 1a) within the MTMS solution had been completed. On the other hand, the methanol generated in Scheme 1a might evaporate from the beaker during the hydrolysis process (plastic wrap could not completely seal the MTMS solution) into the air, causing a continuous decrease in the methanol content detected by the GC measurements. Therefore, the conversion rate of -OCH_3_ reached the maximum value at a *t*_h_ of 30 min.

Each methoxy group (-OCH_3_) of MTMS monomer was immediately hydrolyzed to hydroxyl groups (-OH) under the catalysis of acid in the MTMS solutions [43]. As shown in Figure 2a, the colloidal particle size gradually decreased from ~48.0 nm at the *t*_h_ of 10 min to ~1.0 nm at the *t*_h_ of 1440 min. It indicated that the size of MTMS-derived oligomers changed with the progress of the dehydration reaction of the hydrolyzed MTMS monomers. As the hydrolysis reaction of MTMS solution progressed, under the action of mechanical stirring at 400 rpm, the MTMS-derived oligomers gradually transformed from large MTMS aggregations to several small-sized highly dispersed colloidal particles [44].

The mechanisms behind the formation of MTMS sol were unveiled by their FTIR spectra [45,46]. The characteristic vibration signals displayed in Figure 2b,c were associated with the species arising from the hydrolysis of MTMS at various *t*_h_. The FTIR spectra profile of sol Samples 7–11, 2, and 12–14 were similar. The MTMS sol samples and pure MTMS (see Figure 2b) both had a C-H vibration peak (1270 cm^−1^). However, the Si-C (~1190 cm^−1^), Si-O (~1077 cm^−1^), rCH_3_ (~789 cm^−1^), and C-H (~2843 cm^−1^) characteristic absorption peaks of MTMS (see Figure 2c) disappeared in the MTMS sol samples. We noted that the disappearance of the peak at ~789 cm^−1^ was related to that at ~2843 cm^−1^ [47,48]. The C-H symmetric stretching vibration of MTMS located at ~2941 cm^−1^ shifted to ~2971 cm^−1^ in the MTMS sol samples. Compared to the MTMS, MTMS sol samples showed a significantly decreased intensity in the Si-O-C stretching band, which switched from ~836 cm^−1^ to ~849 cm^−1^. Moreover, the asymmetrical Si-O-Si vibrations led to broad absorption peaks of ~1104 and ~1020 cm^−1^, which are assigned to insoluble silica derivatives. The observed changes in these peak patterns indicated that the MTMS in the OA aqueous solution has been hydrolyzed to varying degrees in different samples [49]. Moreover, the low absorption peak positioned at ~912 cm^−1^ of sol Samples 7–10 arose from considerable amounts of silanols (Si-OH) in T^2^ species (i.e., CH_3_Si(OSi)_2_(OCH_3_) or CH_3_Si(OSi)_2_(OH)), indicating that more dense Si-OH groups appeared at the colloidal particles when *t*_h_ was 10–60 min [50,51]. Longer *t*_h_ (>60 min) would cause the condensation between Si-OH, the constitution of Si-O-Si bonds (~1104 cm^−1^), and the colloidal particle growth, which was consistent with the colloidal particle sizes presented in Figure 2a.

### 2.2. Transparency and Gelation Time (GT) of MTMS Sols

**Figure 3:**
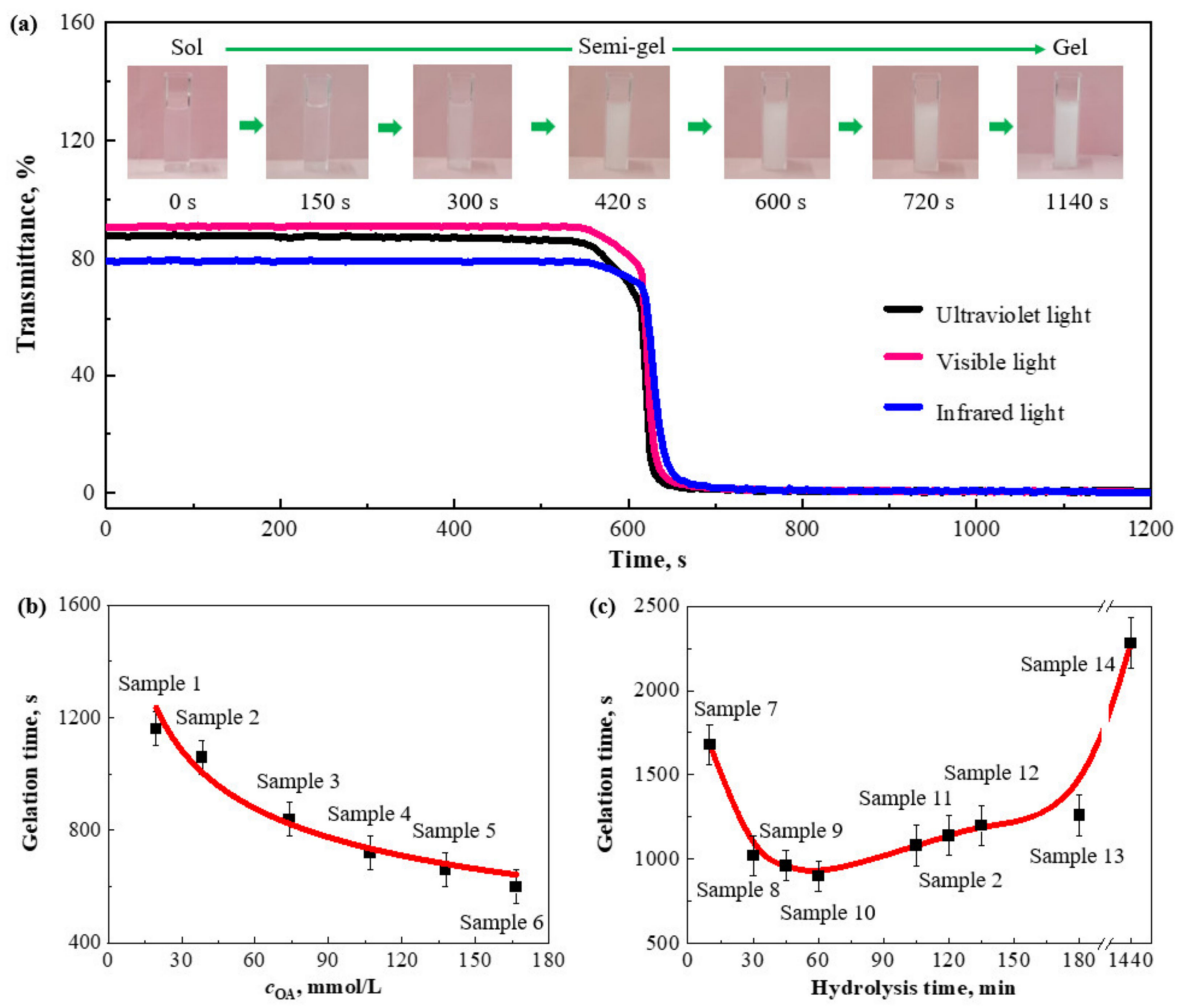
(**a**) Photos of sol Sample 2 during the gelation process and its ultraviolet, visible, and infrared light transmittance with time. Influence of (**b**) *c*_OA_ and (**c**) *t*_h_ on the gelation time of MTMS sol.

The dominant form of the sol-gel process under the basic conditions is the condensation reactions between silanols (Si-OH) [52]. Henceforth, the number of Si-O-Si bonds grew while Si-OH moieties decreased with the gelation progress since the addition of ammonia. The color of the MTMS sol (see Figure 3a) converted from transparent to cloudy and translucent and then to opalescent and opaque during the gelation process. In the initial stage, the sol Sample 2 without ammonia was primarily transparent to visible (~91.0%), ultraviolet (~87.9%), and infrared (~79.6%) light. Subsequently, the transmittance of sol Sample 2 showed a slight change within ~550 s after the addition of ammonia, then declined slowly within 550–600 s, then decreased rapidly at 600–650 s, and finally decreased to ~0% (<1.0%) at 730–770 s. The transmittance of sol Sample 2 could not be reduced to 0%, even when reaching the gelation state (1140 s), but fluctuated in the range of 0–1%. The phenomenon was related to the surface hydroxyl (-OH) content of colloidal particles. As shown in Figure 1, the OA acid catalysis in the MTMS solution could not hydrolyze all the -OCH_3_ on the MTMS to -OH. Similarly, the Si-OH moieties could not all be transformed into Si-O-Si bonds under the catalysis of ammonia. Therefore, the freshly formed gel network possessed numerous Si atoms terminated with -OH and -OCH_3_ groups, indicating that the formed dissociative clusters were not yet linked to the spanning Si-O-Si skeleton network [53]. The gel microstructures could also evolve with the condensation reactions even after the gelation point, which in turn caused the further reduction and fluctuations of the transmittance of sol Sample 2.

The ratio of the number of Si-O-Si bonds that have been formed to those that can be theoretically formed changed over time, relying on the initial concentration of reagents, temperature, and catalysis environment [53]. The GT of MTMS sols (see Figure 3b) decreased significantly from ~1160 s to ~600 s as the *c*_OA_ increased from 19.6 mmol/L to 166.7 mmol/L. This finding was caused by the following two reasons. On one hand, the increased dose of *c*_OA_ increased the water content in the MTMS sol as the OA aqueous solution contained 99.87 wt.% deionized water. The high-water content in MTMS sol could accelerate the hydrolysis process of MTMS solution and generate a high content of Si-OH in MTMS sol, acting as sources for the positive reactions (i.e., condensation reactions) shown in Scheme 1b,c. The gelation rate of MTMS sols was consequently accelerated. On the other hand, the hydrolysis rate increased with increasing *c*_OA_, which led to a faster condensation rate after the addition of ammonia [37].

In comparison, the GT of MTMS sols (see Figure 3c) decreased dramatically from ~1680 s to ~900 s when the *t*_h_ increased from 10 min to 60 min while it increased significantly from ~900 s to ~2280 s when the *t*_h_ further increased to 1440 min. The product microstructure during the sol-gel process evolved continuously with the progress of hydrolysis and condensation reactions [26]. Thus, compared to *c*_OA_, the effects of varying *t*_h_ on the GT were more complex. It should be noted that fluctuations in temperature and humidity and a slight mechanical disturbance may prevent the formation from MTMS sol to wet gel or significantly increase the GT. Therefore, even turning on or off the air conditioning or moving the sol sample in the laboratory may strongly affect the actual GT. Although the GT of the MTMS sol after a certain hydrolysis time did not grow or decline anonymously with the hydrolysis time, the GT could only fluctuate within a small range of ~150 s of a certain value (as shown in Figure 3c). It indicated that once the laboratory environmental factors were well-controlled, there was a certain correlation between the gelation time and hydrolysis time of the MTMS sol. This correlation might be related to the hydrolysis degree of MTMS precursor solutions (i.e., the number of Si-OH and Si-OCH_3_ groups), which was discussed in detail in Section 2.1 and Section 2.3.

### 2.3. Microstructure of As-Synthesized MSQ Aerogels

**Figure 4:**
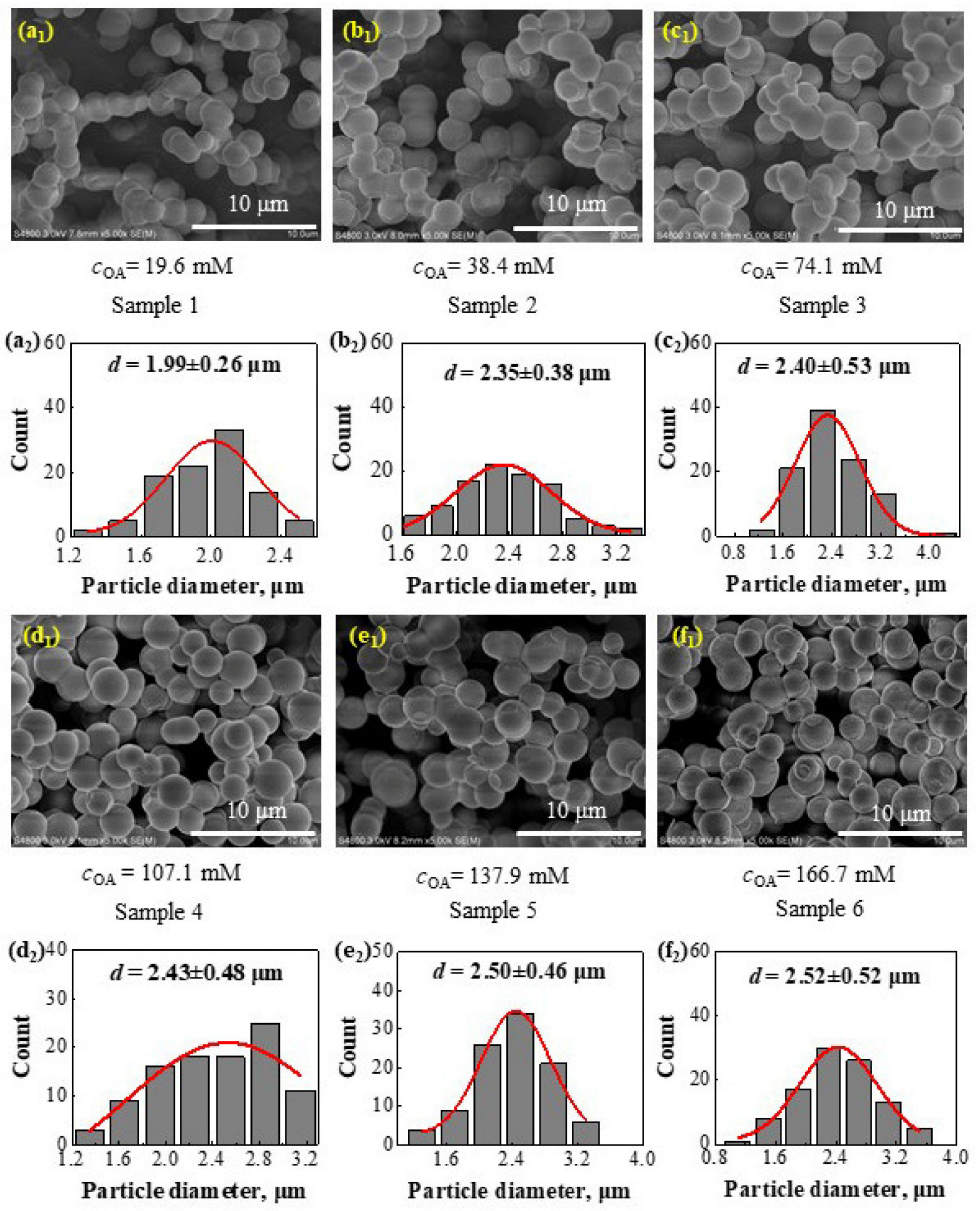
Effect of *c*_OA_ on the (**a_1_**–**f_1_**) microstructure and (**a_2_**–**f_2_**) particle size distributions and average particle size of as-synthesized MSQ aerogel samples.

All the MSQ particles within the aerogel Samples 1–6 (see Figure 4(a_1_–f_1_)) were spherical and were linked by the narrow interparticle linkages, exhibiting the “pearl necklace-like” microstructures. The mean particle size (*d*) of aerogel Samples 1-6 (see Figure 4(a_2_–f_2_)) was 1.99 ± 0.26 μm, 2.35 ± 0.38 μm, 2.40 ± 0.53 μm, 2.43 ± 0.48 μm, 2.50 ± 0.46 μm, and 2.52 ± 0.52 μm, respectively, indicating that *c*_OA_ in the range of 19.6–166.7 mmol/L could slightly increase *d* of the MSQ aerogels. However, compared to previously reported ambient-dried MTMS-derived MSQ aerogels (mean *d* ≤ 100 nm) [40,41], the mean *d* of the as-synthesized MSQ aerogels was much larger. It might be related to the large *h* value (i.e., 17.6–25.2) and small *i* value (i.e., *n*(IPA), *n*(MTMS) = 4.0) in the MTMS sol in this study [20]. A high *h* value indicated a high water content in MTMS sol, which could accelerate the hydrolysis process of the MTMS solution described by Scheme 1a and generate a high content of Si-OH in MTMS sol. The condensation reactions (see positive reactions of Scheme 1b,c) could, in turn, be accelerated. Due to the use of IPA instead of methanol as the solvent for MTMS and the relatively low content of IPA in MTMS sol in this work, the reverse reactions in Scheme 1b would not be significantly suppressed. Numerous Si-O-Si bonds would be continuously generated as the reactions progress. According to the Ostwald ripening mechanism [54], the colloidal particles in this series of experiments grew rapidly in the gelation process, causing the formation of coarse particles in the as-synthesized MSQ aerogels.

**Figure 5:**
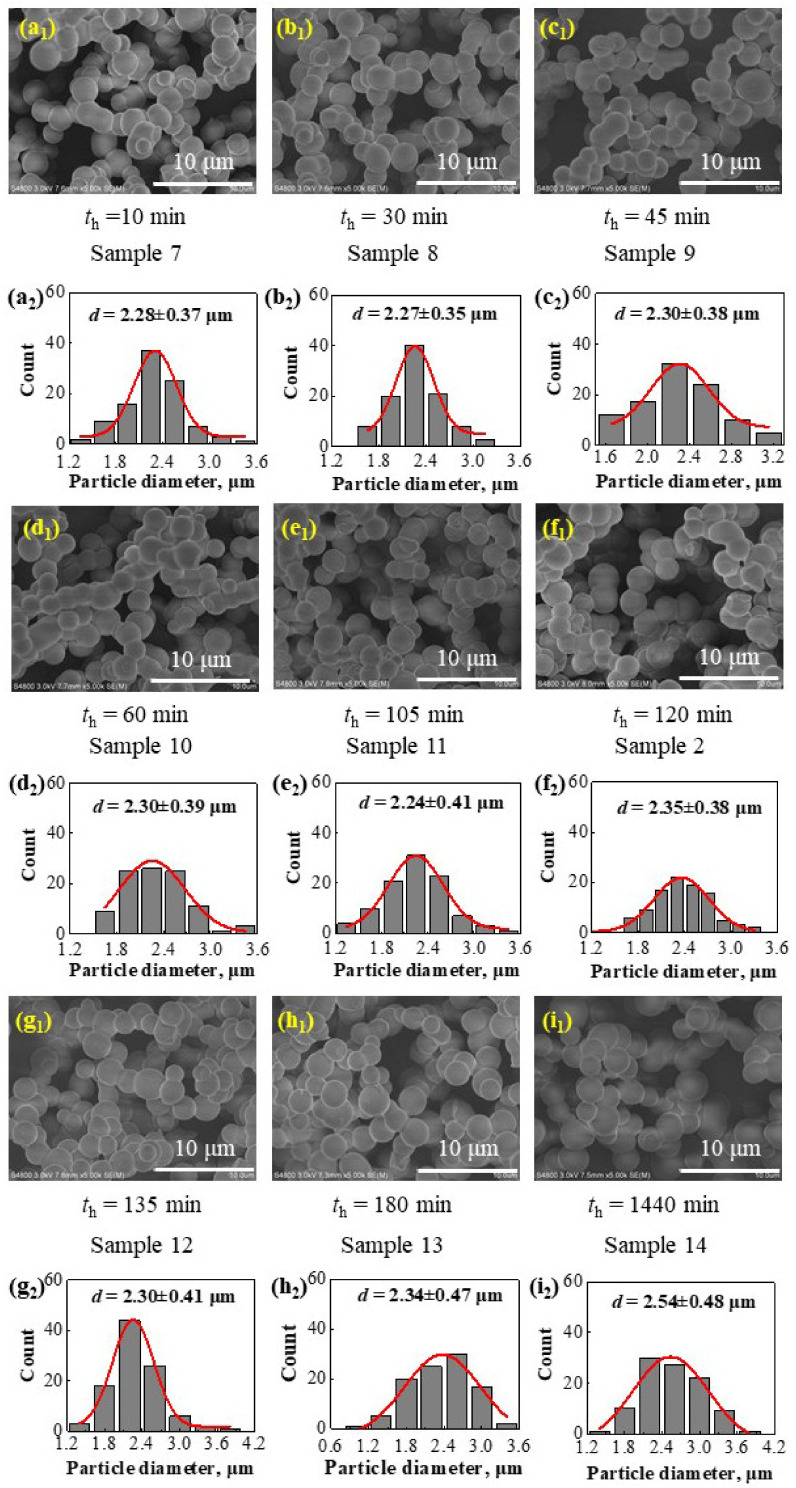
Effect of hydrolysis time of MTMS solution on the (**a_1_**–**i_1_**) microstructure and (**a_2_**–**i_2_**) particle size distributions and average particle size of as-synthesized MSQ aerogel samples.

Similarly, all the silica particles within the aerogel Samples 7–14 (see Figure 5(a_1_–i_1_)) were also spherical and loosely linked, exhibiting inherent “pearl necklace-like” microstructures. There was little difference between the particle sizes (2.24–2.54 μm, see Figure 5(a_2_–i_2_)) within the aerogel samples.

**Figure 6:**
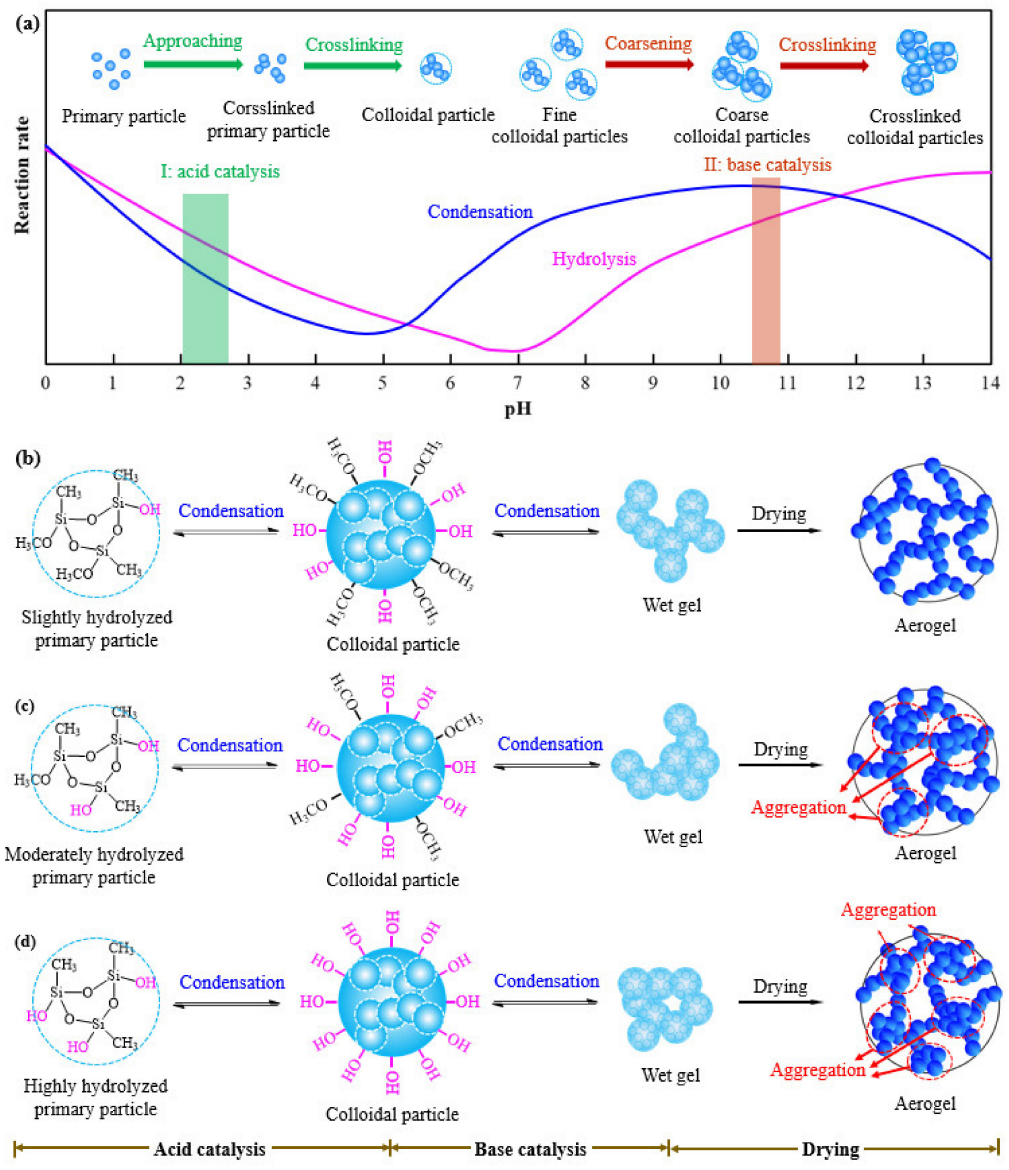
(**a**) Diagram of relative hydrolysis and condensation reaction rates of a silica precursor versus solution pH (reproduced from Ref. [56]) and particle growth and crosslinking mechanism in two-step acid/base catalytic sol-gel process. Schematic diagram of aerogel microstructure formed by (**b**) slightly, (**c**) moderately, and (**d**) highly hydrolyzed primary particles during the two-step acid/base catalytic sol-gel and drying process. Notes: (**a**) is drawn based on the sol-gel kinetics of tetraethylorthosilicate (TEOS). Due to the presence of methyl groups directly bonded to Si atom, MTMS may exhibit biased reaction rates at different pH values (there is no relevant data in the existing literature, and we do not have experimental testing conditions).

As shown in Figure 6a, condensation is the rate-determining reaction, as the hydrolysis reaction possesses a much faster rate under pH in the range of 2.00–2.65. The primary particles first approached and then crosslinked to form colloidal particles. The ripening process is no longer present due to the low solubility of silica in the acidic condition. Therefore, the colloidal particle size growth terminated at 1.0–48.0 nm (see Figure 2a). In contrast, condensation is favored under pH in the range of 10.45–10.70 while hydrolysis is the rate-determining reaction [55]. The hydrolyzed species were easy to be consumed through the condensation reactions.

The surface of slightly (see Figure 6b), moderately (see Figure 6c), and highly (see Figure 6d) hydrolyzed primary particles contained various amounts of -OH groups. Under the weak hydrolysis conditions (i.e., *c*_OA_ < 38.4 mmol/L or *t*_h_ < 60 min), only a portion of the -OCH_3_ group was hydrolyzed to -OH groups. Therefore, the colloidal particle skeletons formed by the -OH crosslinking were relatively loose, and the neck regions of the particles were less connected. In contrast, under moderate hydrolysis conditions (i.e., 38.4 mmol/L ≤ *c*_OA_ ≤ 74.1 mmol/L or 60 min ≤ *t*_h_ ≤ 120 min), more -OCH_3_ groups were hydrolyzed to -OH, leading to a growing number of particle aggregations in the particle skeleton. Moreover, the formed silica particle-to-particle network tent to have a highly branched microstructure under the catalysis of ammonia after the hydrolysis at moderate conditions. Therefore, in the case of a small amount of particle aggregations, the aerogel framework was strengthened to a certain extent, which can resist the shrinkage from the condensation and APD process, thus maintaining a relatively complete skeleton structure and low density. Under the strong hydrolysis conditions (*c*_OA_ > 74.1 mmol/L or *t*_h_ > 120 min), almost all the -OCH_3_ groups could be converted into -OH groups, providing more active sites for the particle coarsening and crosslinking, and making the particle aggregation more obvious. However, at the same MTMS concentration, after the sol-gel process, the volume proportion of the Si-O-Si skeleton could not be changed due to changing the acid catalyst concentrations. Therefore, in the case of a large number of particle aggregations, there would inevitably be many slender skeletons that were difficult to withstand the capillary forces during the ambient drying, resulting in a greater linear shrinkage of the as-synthesized MSQ aerogels.

### 2.4. Optimization of Oxalic Acid Concentration (c_OA_) in the MTMS Precursor

#### 2.4.1. Macroscopic Morphology, Bulk Density, and Linear Shrinkage Ratio of As-Synthesized MSQ Aerogels

**Figure 7:**
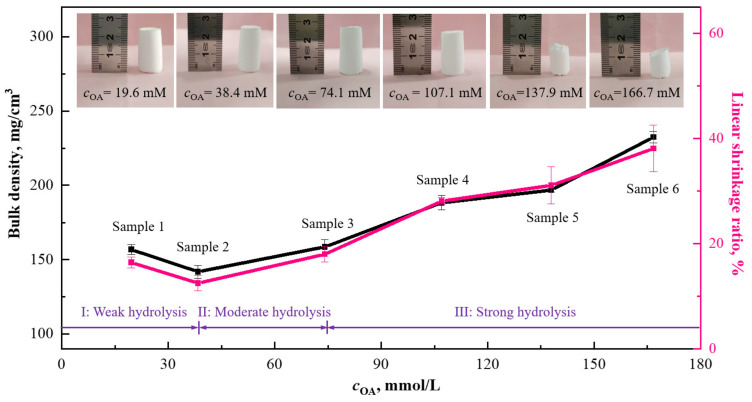
Effect of *c*_OA_ on the macroscopic morphology, bulk density, and linear shrinkage ratio of as-synthesized MSQ aerogels.

As shown in Figure 7, although all wet gel samples were formed in the same cylindrical mold (i.e., *Φ*15 mm) and dried under the same conditions (12 h at 80 °C), their macroscopic morphology varied significantly. Aerogel Samples 1–4 were complete, without any cracks and noticeable size differences between the upper and lower parts. In contrast, although aerogel Samples 5 and 6 were monolithic, some parts of the wet gel peeled from their overall structures (i.e., uneven top and bottom) during the drying process. In addition, the shape of aerogel Sample 6 was curved, indicating that the upper and lower parts shrunk differently. It meant that MSQ aerogel monoliths with complete and consistent morphology from top to bottom could only be synthesized at a *c*_OA_ of 19.6–107.1 mmol/L. Excessive values of *c*_OA_ (≥137.9 mmol/L) would otherwise easily cause defects in the aerogels.

In contrast, the bulk density of as-synthesized MSQ aerogels (see Figure 7) first decreased from 156.8 to 141.9 mg/cm^3^ when the *c*_OA_ value was in the range of 19.6–38.4 mmol/L and then increased significantly to 232.4 mg/cm^3^ when the *c*_OA_ value was in the range of 38.4–166.7 mmol/L. The linear shrinkage ratio of the as-synthesized MSQ aerogels showed a similar trend as *c*_OA_ increased from 19.6 mmol/L to 166.7 mmol/L. This finding can be related to the aggregations of colloidal particles caused by condensation and shrinkage of the wet gel due to the capillary force during the APD process [57,58].

The surface tension and capillary forces created in the wet gel during the evaporation of deionized water and IPA from its inside pores might lead to the linear shrinkage or even pore collapse of the wet gels. Compared with the wet gels with large pores, higher capillary pressures might be generated in the wet gels with small pores, which might damage or even induce the failure of the monolithic aerogel upon ambient drying. Especially for aerogel Samples 4–6, the high value of *c*_OA_ might give rise to the formation of weak chains with uneven particle clusters (see Figure 4(d_1_–f_1_)). Due to the large number of particle aggregations inside, their pore sizes include two types: nanoscale pores within the particle aggregates and microscale pores built by the particle skeleton (see Figure 4). The spherical particles in aerogel Samples 4–6 exhibited a dense three-dimensional structure, with a few pore spaces between the particle skeleton. Moreover, their uneven microstructure made them unsuitable for some slender skeletons inside to withstand the drying pressures, thus producing fragile aerogel structures [37]. Therefore, the wet gel Samples 4–6, with uneven pores size and particle size distributions, easily shrank during the APD process because of the differential and large pressure gradients [59]. Although the repulsive forces between -CH_3_ groups on the colloidal particles could prevent their pores from collapsing, their bulk density and linear shrinkage ratio were as high as 188.3–232.4 mg/cm^3^ and 28.1–38.1%, respectively.

In contrast, the spherical particles in aerogel Samples 1–3 (see Figure 4(a_1_–c_1_)) were linked and formed a three-dimensional structure. Many pores formed by the spherical silica particles could be observed between the particle skeletons. This observation indicates that the high content of Si-OH moieties in the MTMS sol caused by excessive *c*_OA_ (≥107.1 mmol/L) could cause a tight accumulation of colloidal particles, thereby improving the linear shrinkage ratio and bulk density of as-synthesized MSQ aerogels.

#### 2.4.2. Compression Properties of As-Synthesized MSQ Aerogels

**Figure 8:**
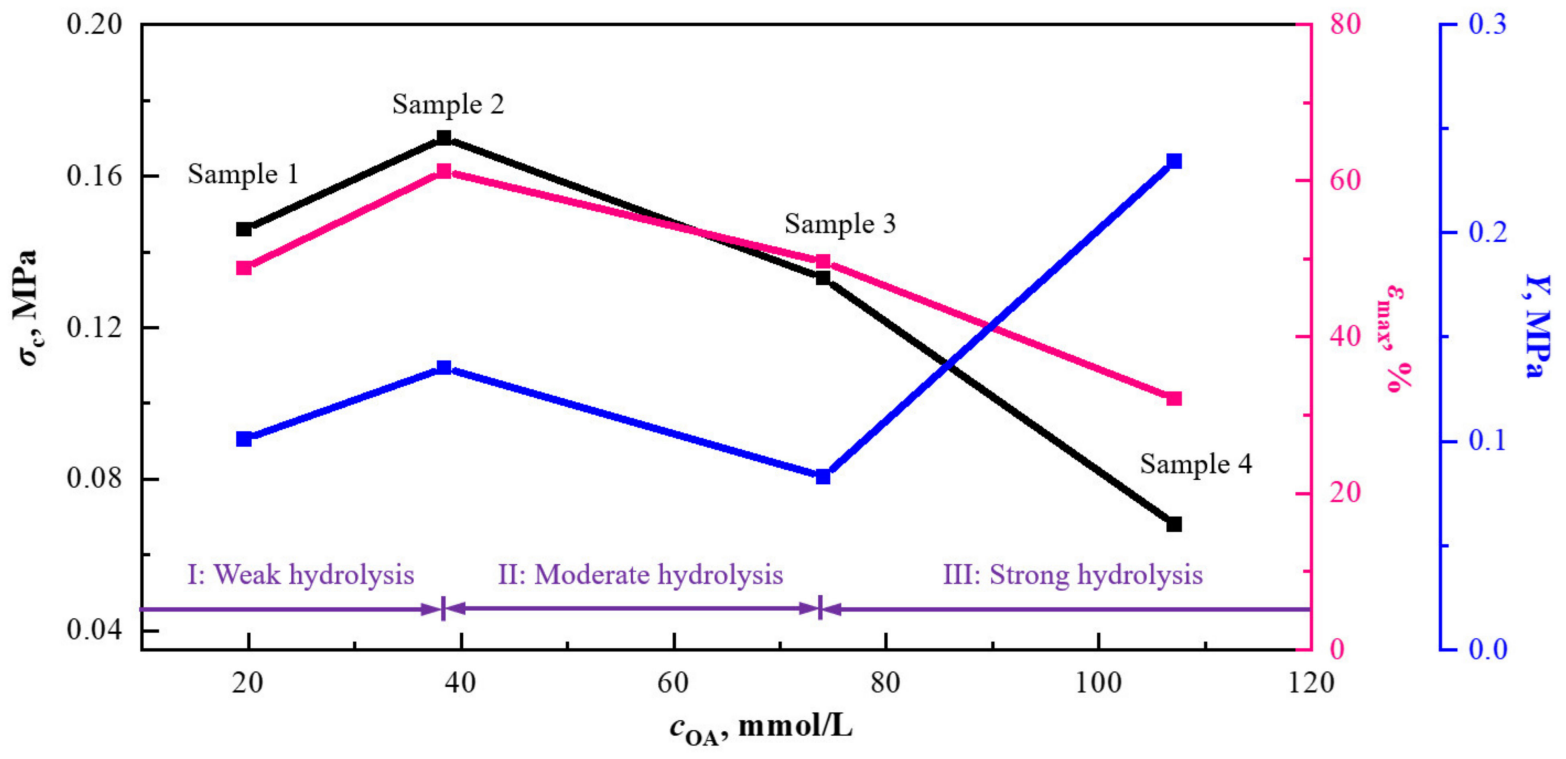
Effects of various *c*_OA_ on the compression strength, maximum compression strain, and compression modulus of as-synthesized MSQ aerogels.

Compared to the compression properties (0.06 MPa, 50%) of MTMS-derived MSQ aerogels synthesized in a CTAB/water/ethanol system [20], the compression strength (0.068–0.170 MPa) and maximum compression strain (32.1–61.2%) of as-synthesized MSQ aerogels (see Figure 8) improved significantly. The high compressibility of the MSQ aerogels could be explained by considering the following three aspects [50]: (1) the enhancement effect of high bulk density (141.9–232.4 mg/cm^3^ in this study compared to 116.0 mg/cm^3^ in the Ref. [20]) on the aerogel skeleton, especially the neck region; (2) Coarser particles constituting the aerogel skeleton might have a smaller probability of deformation and breakage of the neck between the particles, thus experiencing a smaller deformation under the fixed stress [60]. (3) The mutually repulsive interaction of non-polar -CH_3_ on the aerogel skeleton. As *c*_OA_ increased from 19.6 to 166.7 mmol/L, the as-synthesized MSQ aerogels possessed a growing linear shrinkage ratio and increasingly apparent particle aggregation phenomenon (see Figure 4(a_1_–f_1_)). When the *c*_OA_ value was greater than 137.9 mmol/L, numerous microcracks might be generated inside the aerogel due to the high linear shrinkage ratio of the MSQ aerogels (i.e., 31.1–38.1%). Consequently, the compression properties of the aerogels could not be obtained through the universal testing machine. Among aerogel Samples 1–6, aerogel Sample 2 showed the highest compression strength of 0.170 MPa and the maximum compression strain of 61.2%, which was 183.3% and 22.4% higher than the corresponding values (i.e., 0.06 MPa and 50%, respectively) reported in Ref. [20], using a similar APD process. All the aerogel samples exhibited a low compression modulus (*Y*) of ≤0.3 MPa, indicating that they possessed a flexible texture. Through the above analysis, aerogel Sample 2 had the lowest density (~141.9 mg/cm^3^), highest compressive strength (~0.170 MPa), maximum compressive strain (~61.2%), and good macroscopic morphology. Therefore, the optimum *c*_OA_ in the MTMS solution was determined to be 38.4 mmol/L.

### 2.5. Optimization of Hydrolysis Time (t_h_) of MTMS Precursor

#### 2.5.1. Macroscopic Morphology, Bulk Density, and Linear Shrinkage Ratio of As-Synthesized MSQ Aerogels

**Figure 9:**
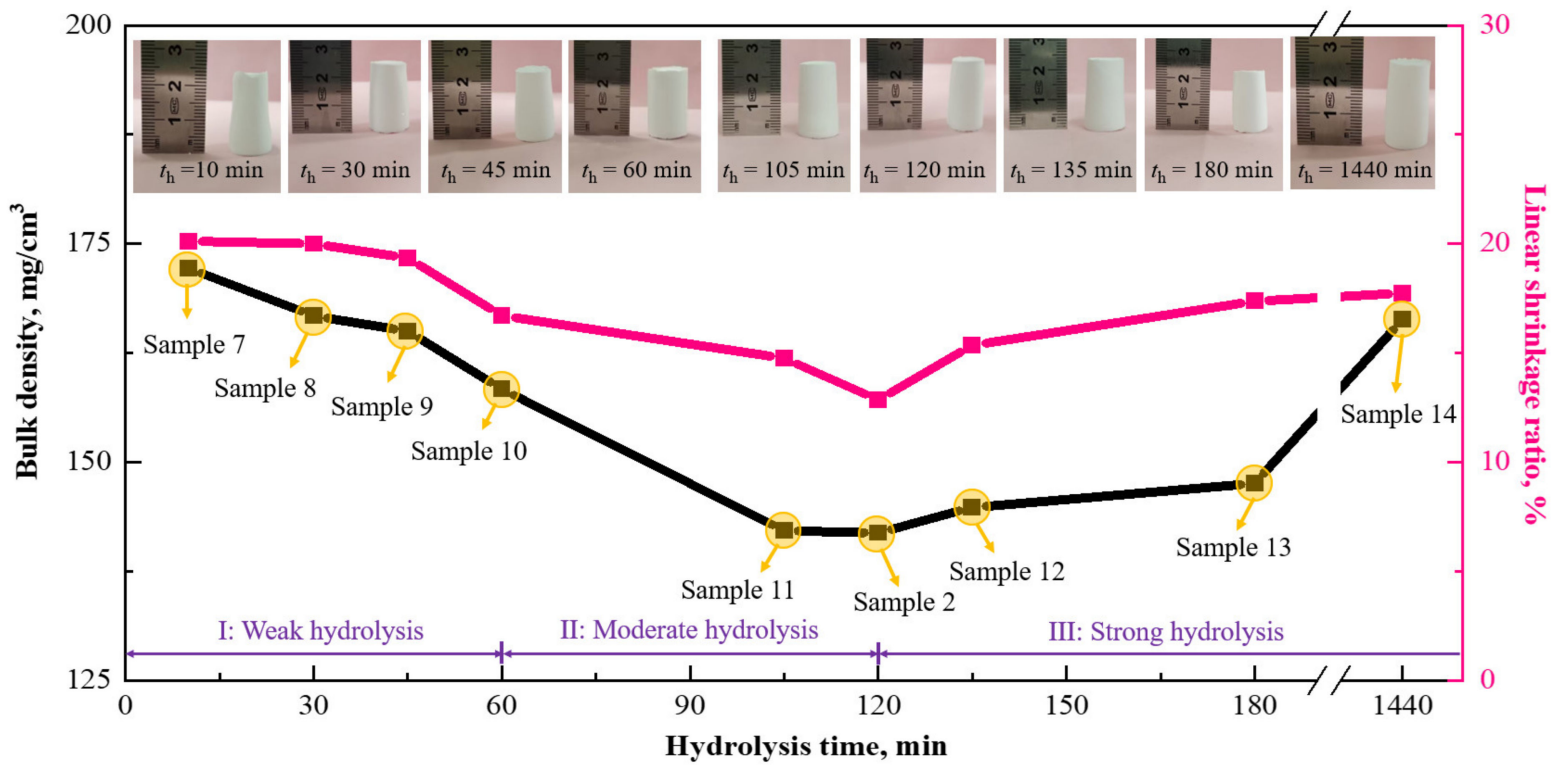
Effect of hydrolysis time (*t*_h_) on the macroscopic morphology, bulk density, and linear shrinkage ratio of as-synthesized MSQ aerogels.

As shown in Figure 9, all the aerogel samples exhibited a complete macroscopic morphology without cracks, but there were noticeable size inconsistencies in the upper and lower parts of aerogel Samples 7, 8, 13, and 14, which were not observed in aerogel Samples 9–12, and 2. Therefore, monolithic MSQ aerogels with intact and consistent macroscopic morphology could only be obtained when *t*_h_ was 60–135 min. Too high (>135 min) or low values (<60 min) of *t*_h_ would easily lead to defects in the as-synthesized MSQ aerogels. Besides, as *t*_h_ increased from 10 to 120 min, the bulk density of MSQ aerogels reduced significantly from 172.2 to 141.9 mg/cm^3^ and their linear shrinkage ratio decreased from 20.1% to 12.9%. However, as *t*_h_ continued to increase from 120 to 1440 min, the bulk density and linear shrinkage ratio of as-synthesized MSQ aerogels no longer decreased, but instead increased from 141.9 to 166.3 mg/cm^3^ and 12.9% to 17.9%, respectively. This finding was related to the growth and crosslinking mechanism of the colloidal particles during the sol-gel process. Under the weak hydrolysis conditions (*t*_h_ < 60 min), as the silica particles were negatively charged and repelled each other, they preferentially grew instead of colliding with each other, without the aggregation of the particles in the gelation process. This in turn led to a poorly Si-O-Si linked inorganic network upon condensation, making it difficult to resist stress during drying, resulting in a significant linear shrinkage ratio and bulk density of the MSQ aerogels [61]. Under moderate hydrolysis conditions (60 min ≤ *t*_h_ ≤ 120 min), the abundant Si-OH on the colloidal particle surface enabled them to aggregate with each other through condensation reactions. The high -CH_3_ concentration on the wet gel surface might produce large repulsive forces, causing a “spring-back” effect of the gel network to resist the linear shrinkage during the APD process [62]. However, under strong hydrolysis conditions (*t*_h_ > 120 min), over-crosslinked particles would aggregate excessively, resulting in a higher linear shrinkage ratio and bulk density of the as-synthesized MSQ aerogels.

#### 2.5.2. Compression Properties of As-Synthesized MSQ Aerogels

**Figure 10:**
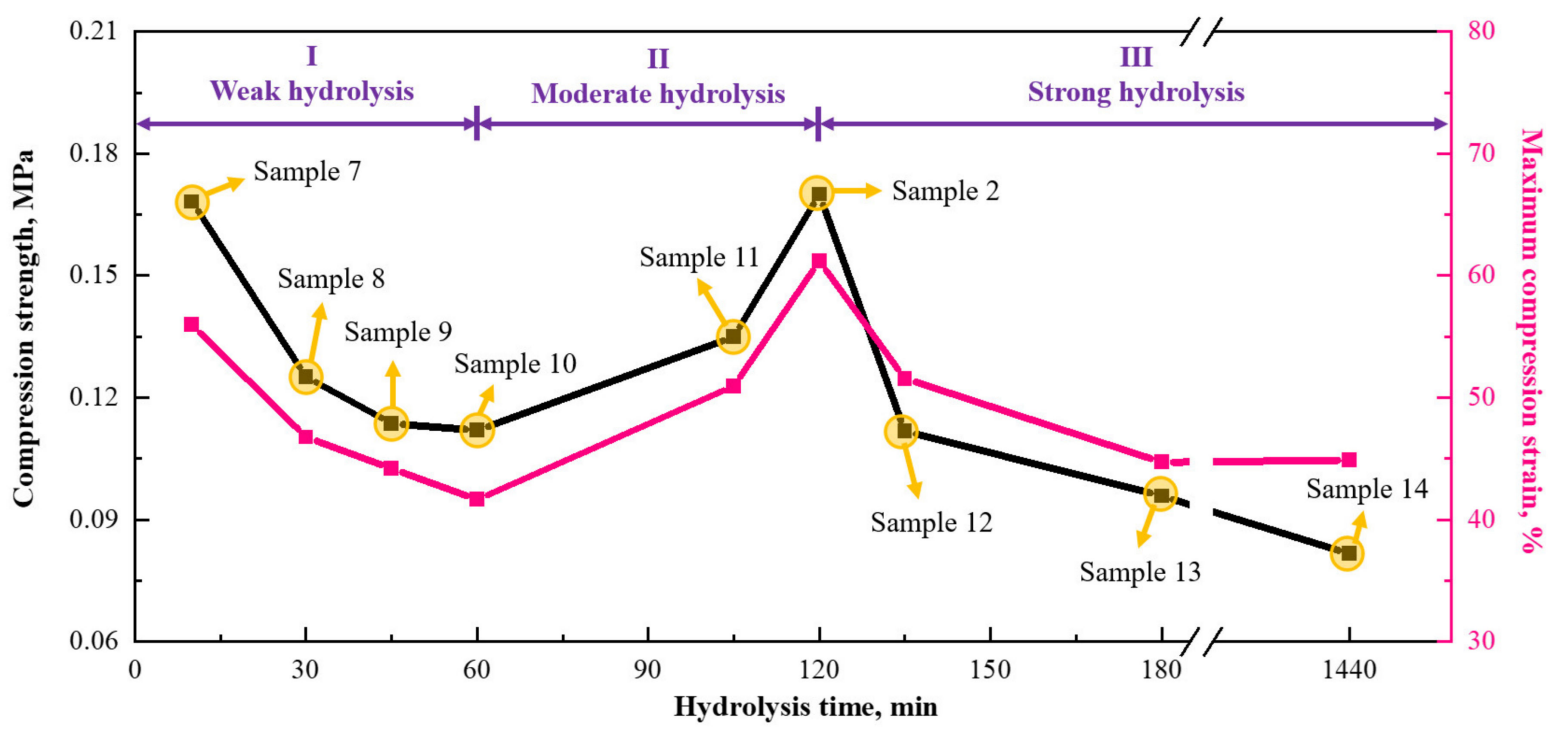
Effect of hydrolysis time (*t*_h_) on the compression strength, and maximum compression strain of as-synthesized MSQ aerogels.

The compression strength-hydrolysis time curve (see Figure 10) falls into three stages: (I) weak hydrolysis conditions (*t*_h_ < 60 min), (II) moderate hydrolysis conditions (60 min ≤ *t*_h_ ≤ 120 min), and (III) strong hydrolysis conditions (*t*_h_ > 120 min). The compression strength of the as-synthesized MSQ aerogels decreased dramatically from an initial high value of 0.168 MPa to 0.125 MPa when *t*_h_ increased from 10 to 45 min, then gradually rose to 0.170 MPa when *t*_h_ further increased to 120 min, and finally significantly fell back to 0.081 MPa. The variations in the compression strength of the as-synthesized MSQ aerogels were related to the microstructure of MSQ aerogels. As shown in Figure 5, the silica network in aerogel Samples 7–9 (*t*_h_ < 60 min) is less connected (i.e., many one-to-one connections), while the particles (*d* = 1.91–2.68 μm) within the network are rather large. In contrast, the silica particles within the aerogel Samples 10, 11, and 2 (60 min ≤ *t*_h_ ≤ 120 min) are well connected (i.e., many one-to-many connections). Although their particle size is around 2.30 μm, the particles could aggregate together to form a thick and strong skeleton. Although further increasing *t*_h_ (>120 min) could make the particle connections denser, the large linear shrinkage ratio (i.e., 15.4–17.7%) of wet gel might cause microcracks among the particle-to-particle connections while weakening the compression strength. The maximum compression strain of the as-synthesized MSQ aerogels exhibited a similar pattern. Therefore, the optimum values of *c*_OA_ and *t*_h_ for the MTMS precursor solution are 38.4 mmol/L and 120 min, respectively. These process parameters endowed the as-synthesized MSQ aerogels with a compression strength of 0.170 MPa and a maximum compression strain of 61.2%. The results of this work thus challenge the common paradigm that silica precursors need to be hydrolyzed for a long time, even to rest overnight, to produce high-quality silica aerogels.

In this paper, all the experimental parameters, including the reagent properties, sol formula, stirring time and rate, lab temperature, and humidity, were fixed to ensure the reliable and reproducible properties of the as-synthesized MSQ aerogels. The root mean square (RMS) error of *σ*_c_ and *ε*_max_ was used to evaluate the reproducibility of compression properties of the as-synthesized MSQ aerogels. The RMS error (see Figure S1 in the Supplementary Materials that adopted the same date from Figure 10) of *σ*_c_ and *ε*_max_ of aerogel Sample 2 (i.e., *t*_h_ deviation = 0 min) that was hydrolyzed for 120 min was less than 0.011 MPa and 4.9%, respectively, which was the minimum among all the samples. However, if *t*_h_ deviation was 15 and −15 min, aerogel Samples 2 and 12 and Samples 2 and 11 were regarded as the same aerogel sample. Their mean *σ*_c_ decreased by 15.9% and 9.4%, respectively, while their mean *ε*_max_ decreased by 7.8 and 8.8% respectively. Moreover, their RMS error of *σ*_c_ was 0.032 and 0.021 MPa, respectively, while the corresponding RMS error of *ε*_max_ reached 6.8% and 6.3%, respectively. A larger *t*_h_ deviation would result in aerogel samples with lower *σ*_c_ or *ε*_max_ and larger RMS errors. Therefore, the reproducibility of compression properties of the as-synthesized MSQ aerogels became poor when *t*_h_ were not controlled accurately. The accuracy of *t*_h_ was crucial for the reproducibility of compression properties of the as-synthesized MSQ aerogels.

## 3. Conclusions

This work addressed a persistent practical challenge to obtain robust polymethylsilsesquioxane (MSQ) aerogels from methyltrimethoxysilane (MTMS) by the common ambient pressure drying method. By implementing an innovative hydrolysis approach, it was revealed that the hydrolysis conditions of MTMS solution had significant effects on the gelation time, linear shrinkage ratio, bulk density, and microstructure of the as-synthesized aerogels. The optimal oxalic acid concentration in the sol and hydrolysis time of MTMS precursor for robust MSQ aerogels were 38.4 mmol/L and 120 min, respectively. To demonstrate the importance of operation under the optimum process conditions, it was found that even a small hydrolysis time deviation of 15 min and −15 min could lead to a 15.9% and 9.4% decrease in the compression strength and a 7.8% and 8.8% decrease in the maximum compression strain of the synthesized aerogel samples, respectively. Furthermore, to obtain MSQ aerogels with stable compression properties, precise control of the acid concentration and hydrolysis time is crucial. Collectively, our findings challenge common paradigms in the synthesis of high-quality silica aerogels and open new avenues for large-scale aerogel production for diverse applications.

## 4. Materials and Methods

### 4.1. Synthesis Method

**Figure 11:**
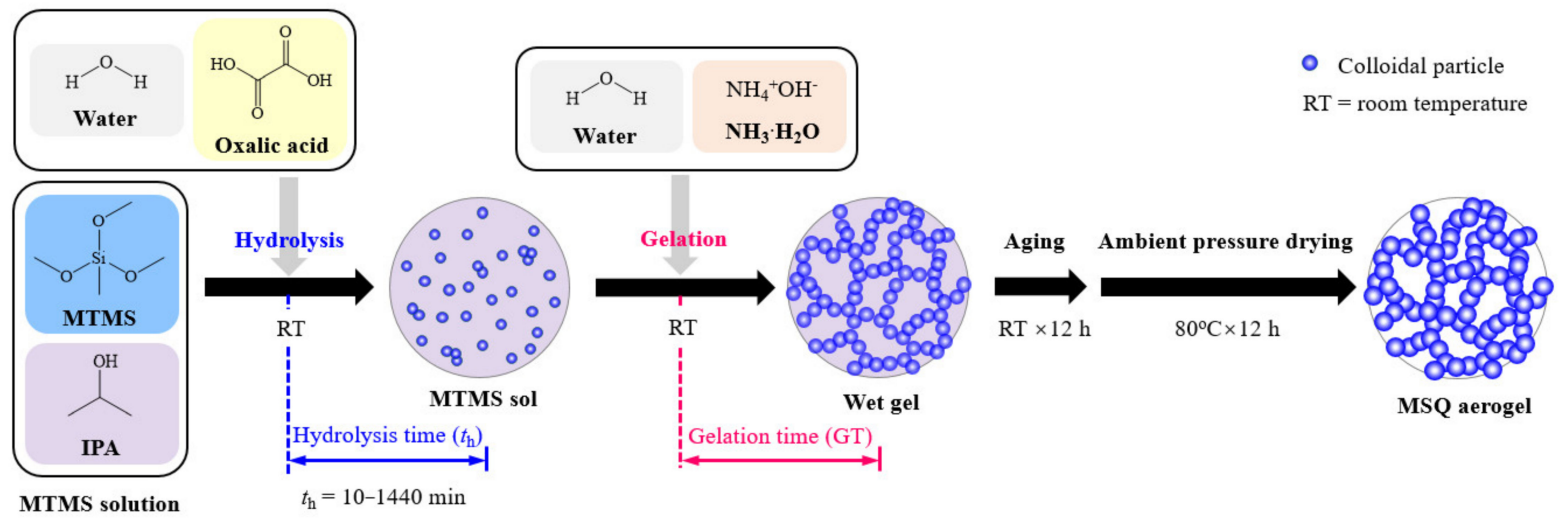
Experimental flowchart for the synthesis of MTMS-derived MSQ aerogels.

In this study, the MTMS-derived MSQ aerogel samples were synthesized by the acid/base two-step catalyzed sol-gel and APD process (see Figure 11). Due to the low reactivity of the MTMS precursor in deionized water (pH = ~7.0), acid and base catalysis were used to increase the reaction rates of hydrolysis and condensation reactions of MTMS precursor. Gelation was initiated in the MTMS precursor systems by pH changes. The required reagents, starting compositions, critical process parameters, and integrity of various MTMS-derived MSQ aerogels are summarized in Table 1 and Table 2, respectively.

First, ethanedioic acid dihydrate (EAD) and pure ammonia water (NH_3_∙H_2_O) were dissolved in deionized water separately to form an oxalic acid (OA) aqueous solution with a 10 mmol/L and NH_3_∙H_2_O aqueous solution with a 4.0 mol/L, respectively. Then, 2.0 mL of MTMS and 4.0 mL of IPA were blended under magnetic stirring for 10 min to form an MTMS solution. Thereafter, the pH of the MTMS solution was adjusted to ~2.5 through the addition of 0.2–2.0 mL as-prepared OA aqueous solution and 4.0 mL deionized water. The MTMS solution was hydrolyzed at room temperature (RT, 20 ± 5 °C) and acidic conditions for various periods (i.e., *t*_h_ = 10, 30, 45, 60, 105, 120, 135, 180, and 1440 min), forming an MTMS sol. Afterward, the pH of the MTMS sol was adjusted to ~10.5 through the addition of 0.9 mL as-prepared NH_3_∙H_2_O aqueous solution. After stirring at 400 rpm for 1 min, 5.0 mL MTMS sol was decanted into an air-tight polypropylene centrifugal tube (*Φ*15 mm) and left to stand at RT and covered with a polyethylene protective film until transformed into a wet gel (dimensions: *Φ*15 mm × 28 mm). MTMS-derived MSQ aerogel samples were synthesized after aging at RT for 12 h and drying at 80 °C for 12 h of the wet gels.

### 4.2. Characterization

The hydrolysis kinetics of MTMS precursor was studied by determining the methanol concentrations produced by MTMS using the gas chromatography (GC) instrument (Nexis GC-2030, Shimadzu, Kyoto, Japan). The hydrolysis degree of MTMS solutions was quantified by the conversion degree of -OCH_3_ (*η*), which was calculated by Equation (1),

*η* = *V*_act_/*V*_theo_ × 100% (1)

where *V*_act_ and *V*_theo_ are the actual and theoretical methanol volumes (i.e., all the −OCH_3_ groups on the MTMS monomer are converted to methanol) generated after the hydrolysis of MTMS solution for some time, respectively.

The pH values of the MTMS precursor were evaluated by a pH meter (Shanghai Yueping Scientific Instrument Co., Ltd., Shanghai, China). In this work, the hydrolysis time (*t*_h_) refers to the time interval from the point of addition of deionized water in the MTMS solution to the point of addition of NH_3_∙H_2_O aqueous solution. The colloidal particle sizes within the MTMS solution at various *t*_h_ were determined by the static and dynamic light scattering instrument (ALV/CGS-3, ALV-Laser Vertriebsgesellschaft m-b.H., Langen, Germany) with a fixed scattering angle of 90°.

The gelation time (GT) of MTMS sol was measured by the elapsed time from the point of addition of the NH_3_∙H_2_O aqueous solution to the moment of loss of bulk fluidity at a 45° tilt. A transmittance tester (LH-221, Shenzhen Lianhuicheng Technology Co., Ltd., Shenzhen, China) was used to record the light transmittances of MTMS sol during the gelation. The wavelengths of visible, ultraviolet, and infrared light emitted from the instrument were 380–760 nm, 365 nm, and 940 nm, respectively.

The linear shrinkage ratio (χ, %) of the as-synthesized MSQ aerogels during the APD process was calculated by Equation (2) [63,64,65]:

*χ* = (*L*_w_ − *L*_a_)/*L*_w_ × 100% (2)

where the *L*_w_ and *L*_a_ are the lengths of the as-synthesized wet gel and MSQ aerogel, respectively. In the linear shrinkage test, a starting MTMS solution of 5.0 mL was decanted into a *Φ*15.0 mm polypropylene centrifugal tube, which was used to prepare a typical cylindrical wet gel sample with a length of ~28.0 mm. Detailed information about the surface chemistry of MTMS, as-synthesized sols, wet gels, and MSQ aerogels was revealed by the Fourier transform infrared (FTIR) spectrometer (Nicolet 6700, Thermo Fisher Scientific, Waltham, MA, USA). The morphology of the as-synthesized MSQ aerogels was investigated by a field emission scanning electron microscope (FE-SEM, S-4800, Hitachi, Tokyo, Japan). A sputtering gold treatment with a duration of 80 s (15 mA, 6.0 E0Pa) was applied to the MSQ aerogel samples before the SEM analysis. The particle size (*d*) within the SEM photos was examined using the Nano measurer 1.2 software.

The axial compression tests of cylindrical MSQ aerogels with ~12 mm diameter and 18–25 mm length were performed with a universal testing system (5967X, Instron, MA, USA). The initial slope from the compression stress-strain curve (i.e., 1–5% strain) was assumed as the compression modulus (*Y*) [66]. The compression strengths (*σ*_c_) and maximum compression strains (*ε*_max_) were assumed at the first noticeable sign of cracking [67]. The root mean square (*RMS*, see Equation (3) error values were used as the evaluation parameter for the compression property stability of the as-synthesized MSQ aerogels [33]:

(3) $$RMS=\sqrt{\frac{\sum_{i=1}^{n}{\left(x_{i}-\bar{x}\right)}^{2}}{n}}$$

where *x_i_* and $\bar{x}$ represent the actual and mean values of *σ*_c_ or *ε*_max_, respectively. The number of aerogel samples (*n*) used to calculate *RMS* for the same starting compositions and same hydrolysis time was 5.

## Figures and Tables

**Table 1:** Reagents for preparing MTMS-derived MSQ aerogels.

Name	Abbreviation	Chemical Formula	Supply Company	Notes
Deionized water	H_2_O	-	Jiangnan University, Wuxi, China	Homemade
Methyltrimethoxysilane	MTMS	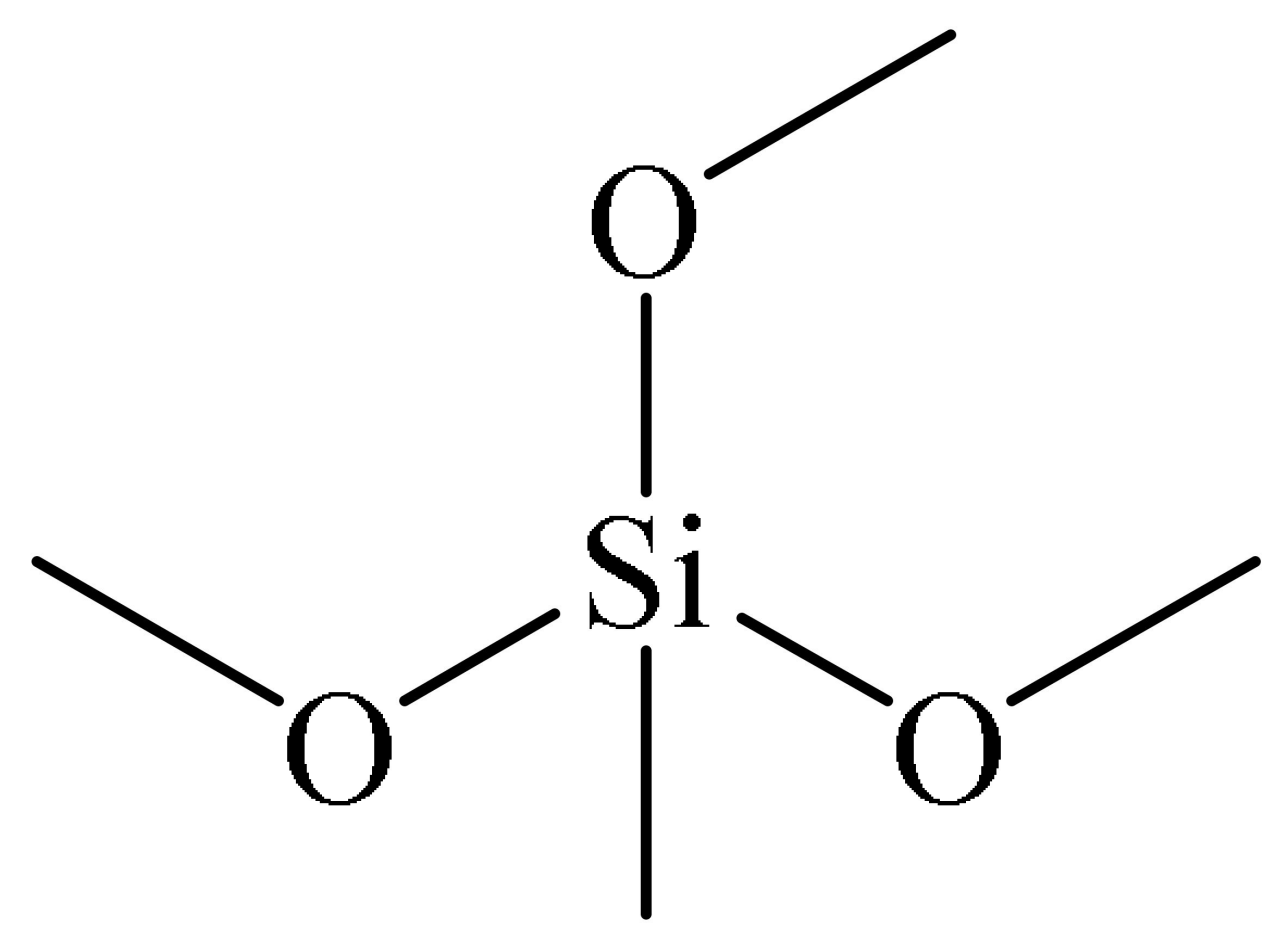	Sinopharm Chemical Reagent Co., Ltd., Shanghai, China	Purity: ≥99.8%
Ethanedioic acid dihydrate	EAD	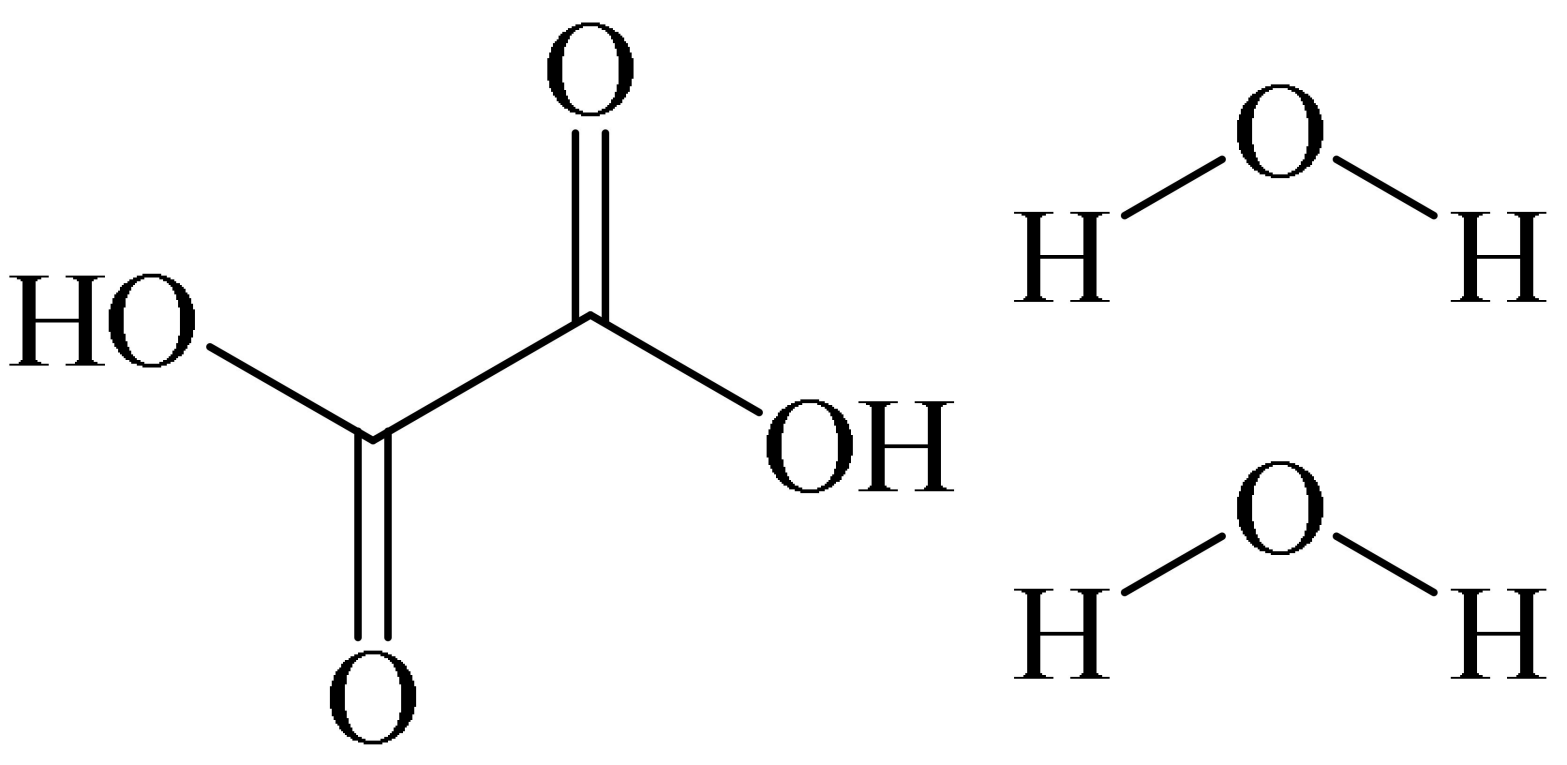	Sinopharm Chemical Reagent Co., Ltd., Shanghai, China	Purity: ≥99.5%
Ammonia water	NH_3_∙H_2_O	NH_4_^+^OH^−^	Sinopharm Chemical Reagent Co., Ltd., Shanghai, China	Ammonia content: 25–28%
Isopropyl alcohol	IPA	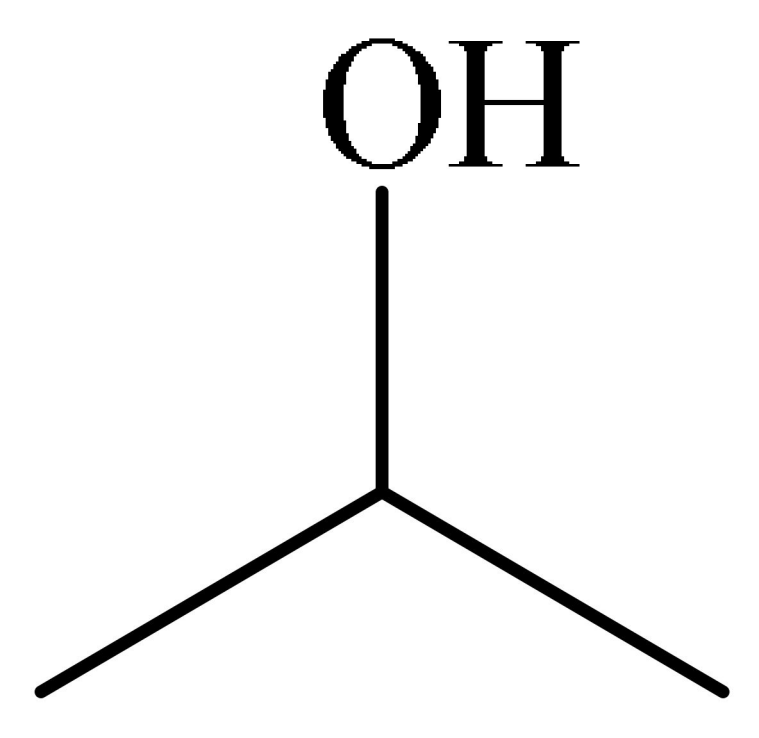	Sinopharm Chemical Reagent Co., Ltd., Shanghai, China	Purity: ≥99.7%

**Table 2:** Starting compositions, critical process parameters, and integrity of various MTMS-derived MSQ aerogels.

Sample	MTMS, mL	IPA, mL	H_2_O, mL	OA Aqueous Solution, mL	*c*_OA,_ mmol/L	*V*_sol_, mL	pH_acid_	*h*	*t*_h_, min	NH_3_·H_2_O Aqueous Solution, mL	pH_base_	Integrity of Aerogel
1	2.0	4.0	4.0	0.2	19.6	10.2	2.55	17.6	120	0.9	10.70	○
2	2.0	4.0	4.0	0.4	38.4	10.4	2.50	18.5	120	0.9	10.55	○
3	2.0	4.0	4.0	0.8	74.1	10.8	2.35	20.2	120	0.9	10.50	○
4	2.0	4.0	4.0	1.2	107.1	11.2	2.20	21.8	120	0.9	10.50	○
5	2.0	4.0	4.0	1.6	137.9	11.6	2.10	23.5	120	0.9	10.45	●
6	2.0	4.0	4.0	2.0	166.7	12	2.00	25.2	120	0.9	10.45	●
7	2.0	4.0	4.0	0.4	38.4	10.4	2.60	18.5	10	0.9	10.55	◐
8	2.0	4.0	4.0	0.4	38.4	10.4	2.60	18.5	30	0.9	10.55	◐
9	2.0	4.0	4.0	0.4	38.4	10.4	2.55	18.5	45	0.9	10.55	○
10	2.0	4.0	4.0	0.4	38.4	10.4	2.50	18.5	60	0.9	10.55	○
11	2.0	4.0	4.0	0.4	38.4	10.4	2.50	18.5	105	0.9	10.55	○
12	2.0	4.0	4.0	0.4	38.4	10.4	2.50	18.5	135	0.9	10.55	○
13	2.0	4.0	4.0	0.4	38.4	10.4	2.45	18.5	180	0.9	10.55	○
14	2.0	4.0	4.0	0.4	38.4	10.4	2.65	18.5	1440	0.9	10.55	◐

Notes: *c*_OA_ = concentration of OA in the sol containing MTMS, IPA, H_2_O, and OA aqueous solution, *V*_sol_ = volume of silica sol containing MTMS, IPA, H_2_O, and OA aqueous solution, pH_acid_ = pH value of the sol containing MTMS, IPA, H_2_O, and OA aqueous solution, *h* = molar ratio of hydrolysis water (including the water in the OA aqueous solution) to the MTMS precursor, i.e., *n*(H_2_O), *n*(MTMS), *t*_h_ = hydrolysis time, pH_base_ = pH value of the sol at ~30 s after the addition of NH_3_·H_2_O aqueous solution. ○: Complete without cracks and noticeable size differences between the upper and lower parts, ◐: complete without cracks but there are noticeable size inconsistencies in the upper and lower parts, ●: fragile structure with cracks or some peeled parts or there are significant curved shapes.

## Data Availability

No new data were created.

## Supplementary Materials

The following supporting information can be downloaded at: https://www.mdpi.com/article/10.3390/gels9090720/s1, Figure S1: Effect of hydrolysis time (*t*_h_) deviation on the compression strength and maximum compression strain of the as-synthesized MSQ aerogels. Notes: the *t*_h_ deviation referred to the maximum difference between the actual *t*_h_ and the optimum *t*_h_ of 120 min. For instance, if the deviation of *t*_h_ was -15 min, *t*_h_ of all aerogel samples could be 120 min and 105 min. If the deviation of *t*_h_ was 60 min, *t*_h_ of aerogel samples could be 120, 135, and 180 min.
