# Supplementary material for: High-Performance Methylsilsesquioxane Aerogels: Hydrolysis Mechanisms and Maximizing Compression Properties
Source: Gels. 2023 Sep 5;9(9):720. doi: 10.3390/gels9090720 (PMC10530417; doi:10.3390/gels9090720)
Supplement: Supplementary file 1 [file gels-09-00720-s001.zip › gels-2566899-supplementary.pdf]

gels

Supplementary Materials

High-Performance Methylsilsesquioxane Aerogels: Hydrolysis Mechanisms and Maximizing Compression Properties

Guihua Zhang [1,2], Chengdong Li [1,2,*], Yuxiang Wang [1,2], Liangliang Lin [1,2] and Kostya (Ken) Ostrikov [3]

1 Key Laboratory of Synthetic and Biological Colloids, Ministry of Education, School of Chemical and Material Engineering, Jiangnan University, Wuxi 214122, China;
2 International Joint Research Center for Photoresponsive Molecules and Materials, School of Chemical and Material Engineering, Jiangnan University, Wuxi 214122, China;
3 School of Chemistry and Physics and QUT Centre for Materials Science, Queensland University of Technology (QUT), Brisbane, QLD 4000, Australia.
* Correspondence: li690612564@126.com

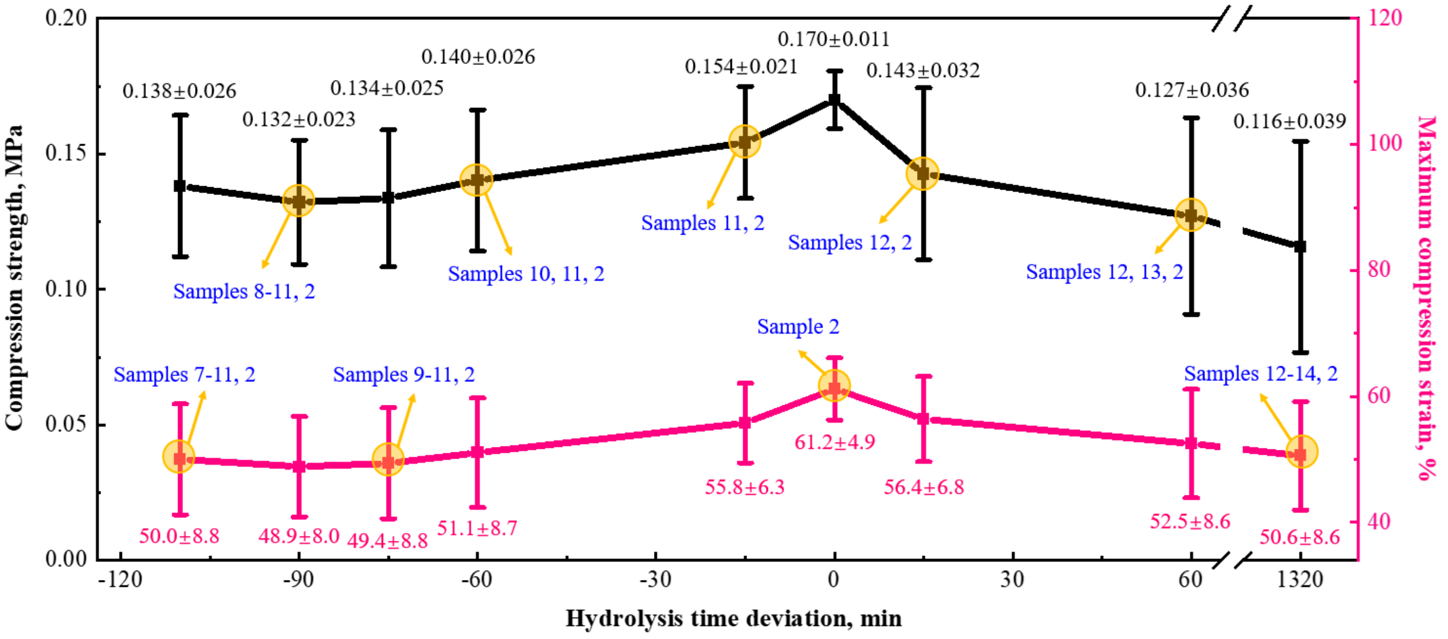

Figure S1. Effect of hydrolysis time (t_h) deviation on the compression strength and maximum compression strain of the as-synthesized MSQ aerogels. Notes: the t_h deviation referred to the maximum difference between the actual t_h and the optimum t_h of 120 min. For instance, if the deviation of t_h was -15 min, t_h of all aerogel samples could be 120 min and 105 min. If the deviation of t_h was 60 min, t_h of aerogel samples could be 120, 135, and 180 min.
